# The complete mitochondrial genome of Black Drongo *Dicrurus macrocercus* (Passeriformes, Dicruridae)

**DOI:** 10.1080/23802359.2020.1827995

**Published:** 2021-11-22

**Authors:** Yiling Fei, Senlin Hou, Yongwu Zhou, Xiaoming Xue

**Affiliations:** Key Laboratory of Wildlife Evidence Technology State Forest and grassland Administration, Nanjing Forest Police College, Nanjing, PR China

**Keywords:** *Dicrurus macrocercus*, mitochondrion genome, phylogenetic analysis

## Abstract

The Black Drongo (*Dicrurus macrocercus*) is an important beneficial bird widespread in China and other Asian countries. In this study, a complete mitochondrial genome of *D. macrocercus* has been obtained by polymerase chain reaction method for the first time. This circular molecule is 17,017 bp in length with A + T contents of 56.9% and contains 2 rRNA genes, 13 protein-coding genes, and 22 tRNA genes. Phylogenetic analysis shows that *D. macrocercus* is genetically closest to *D. hottentottus*.

Black Drongo (*Dicrurus macrocercus*) is a medium sized passerine bird native to much of southern Asia and parts of Indonesia (Zheng 2016). This study is the first report of the complete mitochondrial genome of *D. macrocercus*. The sample was collected from Xinyang City, Henan Province, China (N39°54′, E116°23′). The muscle sample collected from the individual was stored in the Forest Police Forensic Center of State Forestry Administration (Accession S2019J120656001) and the total DNA was isolated by the standard phenol–chloroform extraction procedure (Sambrook and Russell 2001). The complete sequence of *D. macrocercus* mitochondrial genome was determined using L-PCR and conserved primer-walking approaches.

The result showed that the complete mitochondrial genome of *D. macrocercus* is a closed-circular molecule of 17,017 bp in length (GenBank accession No. MT078738). The nucleotide is composed of 32.0% for A, 14.4% for G, 24.9% for T and 28.7% for C, with A + T contents of 56.9%. It exhibited the typical mitogenome structure of birds (Boore 1999; Li et al. 2016; Sun et al. 2016), including two ribosomal RNA genes (rrnL and rrnS), 13 protein-coding genes (PCGs), 22 transfer RNA genes (tRNAs), and a non-coding control region. The 22 tRNA genes cover all 20 standard amino acids and range in size from 66 to 75 bp. The 13 conserved PCGs include 7 subunits of NADH dehydrogenase (NAD1, NAD2, NAD3, NAD4, NAD4L, NAD5, and NAD6), 3 subunits of cytochrome c oxidase (COX1, COX2, and COX3), 2 subunits of ATPase (ATP6 and ATP8), and apocytochrome b (CytB). Among them, the start codon for COX1 is GTG, while the 12 remaining PCGs used ATG as start codon. The stop codons for 13 PCGs are more diverse as ATP6, ATP8, CytB, NAD3, and NAD4L were terminated with TAA; NAD1 and NAD5 were terminated with AGA; NAD6 was terminated with TAG; COX1 was terminated with AGG; NAD2 was terminated with TA; COX2, COX3, and NAD4 were terminated with a single T.

Phylogenetic analysis was conducted for the entire mitogenome of *D. macrocercus* and 20 other species using the maximum-likelihood (ML) method of MEGA 7.0 with 1000 bootstrap replicates (Felsenstein 1981; Kumar et al. 2016). The phylogenetic tree divided the 21 species into 10 groups (Figure 1). *D. macrocercus* is clustered with *D. hottentottus*, which is in accordance with the traditional morphological classification (Zheng 2016). The phylogenetic analysis indicated that *Dicrurus* spp. were genetically closest to the family Laniidae and Corvidae.

**Figure 1.**
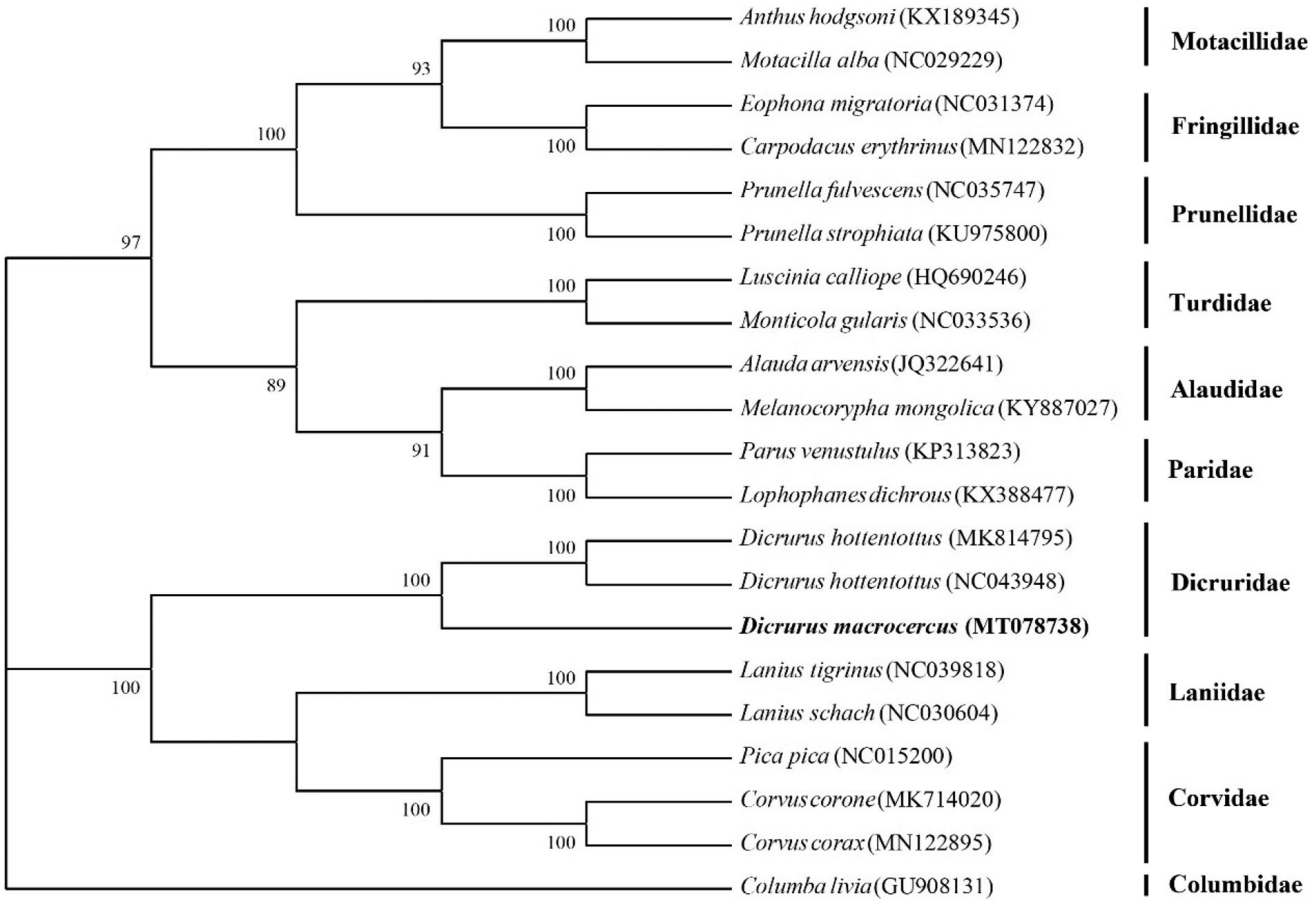
The maximum likelihood phylogenetic tree for *D. macrocercus* and other related avian species. *Columba livia* was used as an outgroup and numbers next to nodes are support values obtained after 1000 bootstrap replicates.

## Data Availability

The data that support the findings of this study are openly available in NCBI at https://www.ncbi.nlm.nih.gov/.
